# T245. THE ROLE OF PROTECTIVE FACTORS IN THE FIRST-EPISODE PSYCHOSIS: PRELIMINARY RESULTS

**DOI:** 10.1093/schbul/sby016.521

**Published:** 2018-04-01

**Authors:** Regina Vila-Badia, Anna Butjosa, Núria Del Cacho, Itziar Riera-López de Aguileta, Mar Alvárez, Marta Pardo, Marta Coromina, Núria Grases, Susana Ochoa, PROFEP Group, Judith Usall

**Affiliations:** 1 Parc Sanitari Sant Joan de Déu; 2 Parc Sanitari Sant Joan de Déu, University of Barcelona; 3 Hospital Sant Joan de Déu; 4 Parc Sanitari San Joan de Dèu, CIBERSAM

## Abstract

**Background:**

Currently, there is a great interest in stress since many diseases can be affected by stress, including psychotic disorders. Interpretation and capacity of the person to tackle situations of psychosocial stress and their recovery capacities are relevant factors in the prevention of psychotic disorders (López-Soler, 2008; N Pereda, 2009, 2010; Noemí Pereda, Guilera, Forns, & Gómez-Benito, 2009). Some of protector factors that have been studied are the following: Resilience (R), Coping Strategies (CS) and Social Support (SS). Furthermore, few studies have been performed with FEP population.

**Methods:**

This research was part of a longitudinal observational study called ‘PROFEP Group’ in Catalonia. The patients belong to Mental Health Parc Sanitari Sant Joan de Déu (for adults) and Hospital Sant Joan de Déu (for children and adolescents) health care sector. Participants were FEP patients (N=15); males= 9, females= 6) and HC (N=19; males=6, females=13) between 14 and 42 years. We used the PANSS scale (positive, negative and general) to evaluate psychotic symptoms and DUKE (social support), EMA (coping strategies) and CD-RISC-17 (resilience) scales to evaluate protective factors.

**Results:**

FEP patients showed worse resilience (p<0.05), less social support (p<0.05) and more avoidance coping strategies (p<0.05) than HC. On the other hand, in FEP patients, some protective factors correlate with the symptomatology. The DUKE scale and the EMA cautious action subscale correlate with the total PANSS, while the EMA social joining subscale correlates with the positive symptoms (p<0.05).

**Discussion:**

Resilience, Coping Strategies and Social Support seem to have an important role in the appearance and severity of an FEP. It is necessary to carry out more studies with more sample, even so, the results indicate that these factors may be important for the prevention of an FEP and could be worked on in future interventions in FEP patients as well as in HC.

